# Wearable Sensor-Based Phase Segmentation Analysis of Front Crawl Swimming: A Scoping Review

**DOI:** 10.3390/s26134221

**Published:** 2026-07-03

**Authors:** Jonathan Simoes, Samuel Aylward, Daniel Hamze, Daniel James Goble, Daniel M. Russell, Joshua Haworth

**Affiliations:** 1 School of Health Science, Oakland University, Rochester, MI 48309, USA; jesimoes@oakland.edu (J.S.); dgoble@oakland.edu (D.J.G.); 2 School of Exercise Science, Ellmer College of Health Sciences, Macon & Joan Brock Virginia Health Sciences at Old Dominion University, Norfolk, VA 23529, USA; dmrussel@odu.edu

**Keywords:** front crawl, swimming, stroke phases, wearable sensors, scoping review

## Abstract

Front crawl swimming stroke phase segmentation has historically relied on video analysis, but the development of wearable sensor technology has created new opportunities for automated phase segmentation. This scoping review mapped the available evidence on wearable sensor-based stroke phase segmentation methods in front crawl swimming, following PRISMA-ScR guidelines. A systematic search of SPORTDiscus, Web of Science, and IEEE Xplore conducted from January to June 2026, identified 15 eligible peer-reviewed studies published between 2000 and 2024. The review revealed an emerging field of research that has converged methodologically around inertial measurement units (IMUs) and the Chollet phase segmentation framework while remaining heterogeneous in algorithmic approach and validation practice. Most notably, no included study reported force or pressure outcomes of any kind, representing a critical gap between current sensor-based segmentation capabilities and the biomechanical information most relevant to applied coaching. Continued work in algorithm validation and intra-phase force measurement is needed to advance the field toward a more complete and practically applicable understanding of front crawl swimming mechanics.

## 1. Introduction

Front crawl, also known as freestyle, is the most commonly trained and competed stroke in the sport of swimming. Extensive biomechanical investigation over the past few decades has established the existence of distinct temporal phases within the front crawl stroke cycle [1,2]. While there remains some discrepancy between stroke phase models, researchers and coaches alike have been able to use these stroke phases to further investigate optimal biomechanics and guide technique-focused coaching decisions.

**Figure 1:**
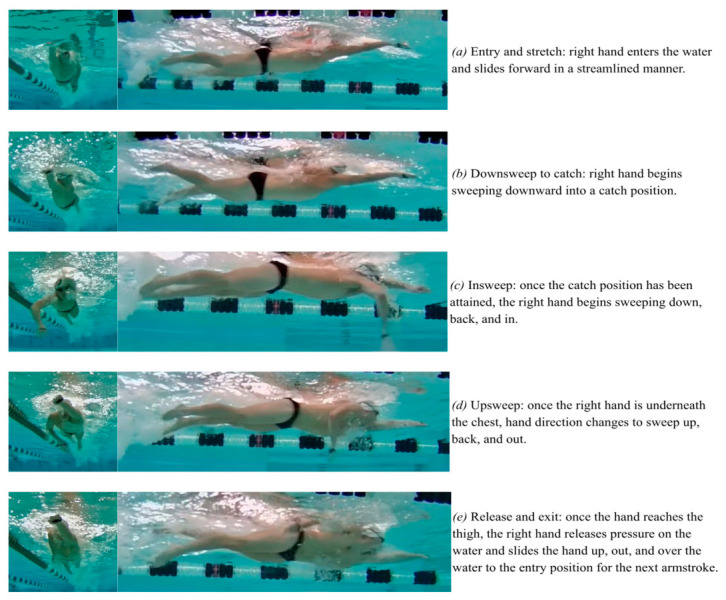
Illustration of the Maglischo [2] freestyle stroke phase segmentation model. Each row depicts the initial frame of each phase of the stroke cycle: (**a**) entry and stretch, (**b**) downsweep to catch, (**c**) insweep, (**d**) upsweep, and (**e**) release and exit. **Left column**: anterior underwater view. **Right column**: lateral underwater view.

**Figure 2:**
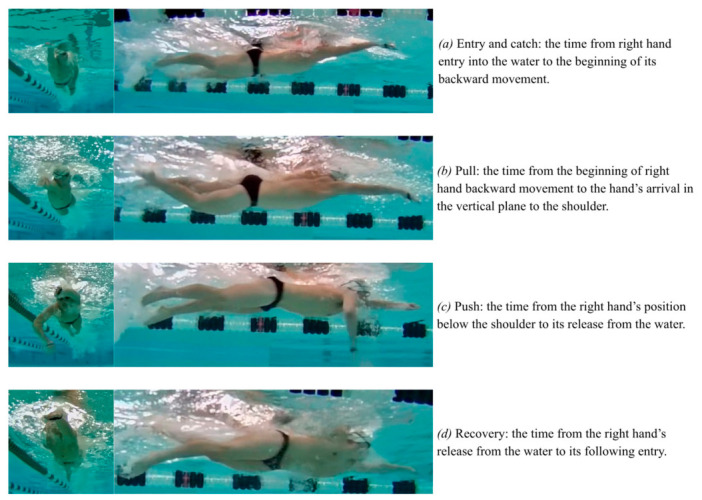
Illustration of the freestyle stroke phase segmentation model as described by Chollet [1]. Each row depicts the initial frame of each phase of the stroke cycle: (**a**) entry and catch, (**b**) pull, (**c**) push, and (**d**) recovery. **Left column**: anterior underwater view. **Right column**: lateral underwater view.

Two primary stroke phase frameworks have been used to segment the front crawl stroke cycle. The first, developed by Ernest Maglischo, divides the stroke cycle into five phases: entry and stretch, downsweep to catch, insweep, upsweep, and release and exit (see Figure 1) [2]. This model emphasizes movement-based definitions as phase events, with each phase boundary defined by a change in direction or pattern of the hand’s path through the water. The other primary framework, developed by Didier Chollet et al., divides the stroke cycle into four phases: entry and catch, pull, push, and recovery (see Figure 2) [1]. This model emphasizes temporal definitions as phase events, with phase boundaries defined by key moments in the arm stroke rather than continuous hand path movement. This model was developed alongside the index of coordination (IdC), a metric that quantifies the temporal relationship between the propulsive actions of each arm [1].

Through the application of these frameworks, researchers have been able to investigate a range of technical and performance-related outcomes in front crawl swimming. At the stroke phase level, phase analysis has enabled the quantification of phase durations and their relative contribution to the overall stroke cycle [1]. As a result, these temporal descriptors have supported the examination of coordination metrics, most notably IdC [1]. Beyond temporal and coordination outcomes, phase segmentation has provided a framework for detailed kinematic analysis, allowing researchers to examine linear velocity, angular kinematics, and hand/wrist movement patterns within phases of the stroke [3,4]. At the performance level, phase-based analysis has also been used to track fatigue-related changes across repeated efforts [5]. One of the most informative contributions in this space was intra-phase force analysis, which has been used to directly quantify the propulsive contribution of each phase of the stroke, providing detailed insight into the mechanical demands of front crawl swimming [3].

Historically, this detailed phase segmentation analysis was only possible via video analysis, a time-intensive process with limited usability in everyday training [6]. However, the advancement of wearable technology has created new opportunities for automated phase segmentation [4]. A growing body of research, addressed in the following sections, has explored the use of wearable sensors to identify stroke phase events, enabling phase segmentation without reliance on video analysis. Despite this growth, the field remains methodologically heterogeneous, as studies differ substantially in sensor type, placement, phase detection algorithm approach, and the technical and performance outcomes calculated from sensor-based phase segmentation.

Prior work has evaluated sensor-based stroke phase analysis across swimming strokes at a broad level [7]. Despite this, no synthesis has systematically mapped the methodological landscape of front crawl phase segmentation, including the specific algorithmic approaches, validation strategies, and technical and performance outcomes calculated from sensor-based segmentation. This gap in available syntheses makes it difficult to identify possible methodological consensus or areas that require further investigation. A scoping review was chosen as the type of review due to its suitability for exploring broad, complex, and heterogeneous bodies of literature.

The objective of the present review is to map the available evidence on sensor-based stroke phase segmentation methods in front crawl swimming. Specifically, this review aims to identify: (1) the types of sensors used and their placement locations; (2) the algorithmic approaches used for segmentation; (3) how previous algorithms have been validated; and (4) the technical and performance outcomes calculated from sensor-based phase segmentation.

The rest of this paper is organized as follows: Section 2 describes the methods used to conduct the scoping review; Section 3 presents the results of the search and data extraction; Section 4 discusses key findings, identifies gaps and limitations, and proposes directions for future research; and Section 5 presents the conclusions.

## 2. Materials and Methods

This scoping review followed the guidelines of the Preferred Reporting Items for Systematic Reviews and Meta-Analyses Extension for Scoping Reviews (PRISMA-ScR) checklist [8] and was managed using Covidence systematic review software (Veritas Health Innovation, Melbourne, Australia; https://www.covidence.org, accessed on 17 June 2026). The complete PRISMA checklist can be found in Supplementary Files S1. This review was retrospectively registered with the Open Science Framework after completion of the analysis (https://doi.org/10.17605/OSF.IO/WMC5P).

### 2.1. Search Strategy

To identify relevant publications, a search string was developed to include terms related to sensor-based phase segmentation analysis of front crawl swimming. Studies were identified through the following methods: (1) electronic databases (SPORTDiscus, Web of Science, and IEEE Xplore), and (2) reference list screening of all studies that passed full-text review. SPORTDiscus and Web of Science were selected as the primary databases for sport and exercise science literature, as the topic was not considered to be of sufficient clinical relevance to warrant searching medical databases. The search of these databases occurred in January 2026. A supplemental search of IEEE Xplore was conducted in June 2026 to capture additional sports engineering literature. The search strategy used keyword searches, accounting for synonyms, related terms, and word variations. The following Boolean search terms were used: “(swim*) AND (front crawl OR freestyle) AND (stroke phase* OR phase segmentation OR arm stroke OR stroke cycle OR propulsive phase* OR pull OR push OR recovery) AND (wearable* OR sensor* OR accelerometer* OR gyroscope* OR IMU OR inertial measurement unit).” Reference list screening was performed by evaluating the reference lists of each included full text.

### 2.2. Eligibility Criteria

To be eligible for inclusion, studies must have: (1) analyzed front crawl/freestyle swimming strokes; (2) employed at least one wearable device; (3) used wearable sensor data to segment the freestyle stroke into phases, regardless of whether additional methods such as video analysis were also employed; (4) included original, empirical research; and (5) been published in English in a peer-reviewed journal. Exclusion criteria consisted of studies that (1) only analyzed non-front crawl/freestyle strokes, or (2) were exclusively simulations of swimming.

### 2.3. Study Selection

All records retrieved from the database searches were imported into Covidence for deduplication and screening. Screening was conducted in two stages: title and abstract screening, followed by full-text review. A review team of three members participated in both stages, with conflicts being resolved through team discussion, and the primary reviewer (first author) making any necessary final decisions.

### 2.4. Data Extraction and Analysis

Following the completion of the screening stages, data extraction was performed. A structured data extraction form was developed a priori and pilot tested using four included studies, with the form refined before full extraction. All included studies, including the pilot testing studies, were subsequently extracted in full. Data extraction was conducted in duplicate, with studies distributed across the three-member review team such that each study was independently extracted by two reviewers. Any discrepancies between reviewers during data extraction were resolved through team discussion, with the primary reviewer making any necessary final decisions. The following variables were extracted from each included study: (1) study metadata (author, journal, year); (2) study purpose; (3) funding sources and conflicts of interest; (4) participant characteristics; (5) study context (environment, pool length, trial length, trial count, trial intensity); (6) sensor utilization (type, brand, sampling rate, placement, number); (7) stroke phase model utilized; (8) algorithmic phase detection and processing methods; (9) phase segmentation algorithm validation; and (10) outcomes reported.

Given the nature of the present scoping review, critical appraisal of individual sources of evidence was not performed. Rather than drawing definitive conclusions, the synthesis of results was structured to systematically capture and present the breadth of available evidence, with the primary aim of mapping the range of evidence and identifying key concepts, common themes, and gaps related to sensor-based phase segmentation in front crawl swimming. Findings are presented through narrative synthesis supported by summary tables.

## 3. Results

### 3.1. Article Selection and Identification

**Figure 3:**
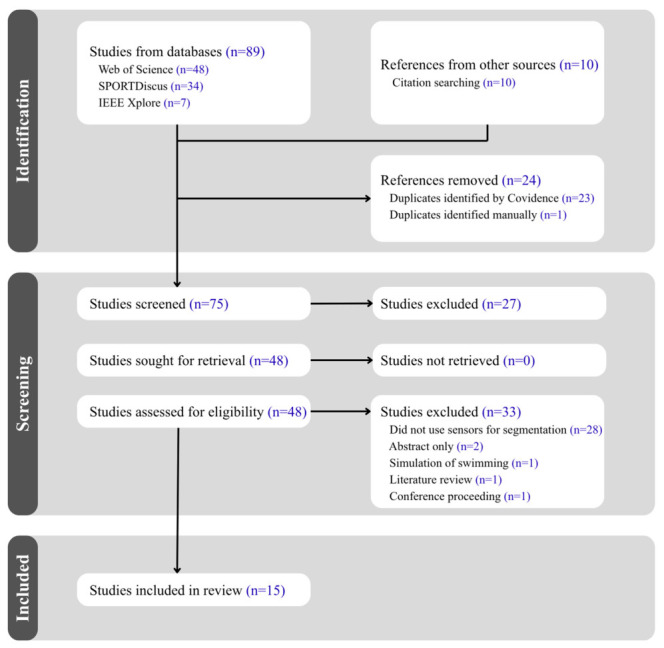
PRISMA-ScR flowchart.

The search strategy returned 89 records across Web of Science (*n* = 48), SPORTDiscus (*n* = 34), and IEEE Xplore (*n* = 7). An additional 10 records were identified through citation searching of included studies. After removing 24 duplicates, 75 records were screened by title and abstract, of which 27 were excluded for lack of relevance. The remaining 48 records were assessed for full-text eligibility, and a further 33 were excluded for the following reasons: 28 did not use sensors for stroke phase segmentation, two were available as abstracts only, one was a simulation of swimming, one was a literature review, and one was a conference proceeding. A total of 15 studies were included in the final review. Results of the search are summarized in Figure 3.

### 3.2. Study Characteristics

The 15 included studies were published between 2000 and 2024, with the majority (*n* = 10, 67%) published within the past decade, suggesting recent growth in the use of wearable sensors for stroke phase analysis in front crawl swimming. These studies represented a varied demographic of athletes, with nine studies (60%) including both male and female participants, and six studies (40%) focusing exclusively on male athletes. Where reported, the age of participants spanned from 15 to 36. National-level athletes were most commonly studied (*n* = 7, 47%), followed by elite, regional, triathlon, and Paralympic levels (*n* = 2 each, 13%), with collegiate and recreational levels each represented in one study (7%). This information can be seen in Table 1. Experimental protocols varied considerably, with some studies using maximal efforts, others submaximal, others race pace, and several using self-selected pace or externally imposed intensities, limiting direct comparability of findings across the literature.

### 3.3. Sensor Characteristics

Sensor characteristics for all included studies are presented in Table 2. The three earliest studies [4,9,10] used two biaxial accelerometers configured to capture triaxial acceleration data, representing the technological constraints of the period. Of the studies to follow, 11 of the 15 (73%) employed IMUs for analysis, with the study from Callaway et al. [11] being the only one to utilize only triaxial accelerometers. Of the 11 studies to include IMUs, eight employed an IMU equipped with a triaxial accelerometer, gyroscope, and magnetometer, with the remaining three studies only including the triaxial accelerometer and gyroscope. The total number of sensors used in the included studies ranged from two to seven. Sensor placement showed a pattern of similar areas, with the forearm as the most common (*n* = 13, 87%), followed by the lower back (*n* = 7, 47%), upper arm (*n* = 6, 40%), hand (dorsal) (*n* = 3, 20%), and thorax/chest (*n* = 3, 20%).

### 3.4. Stroke Phase Segmentation Methods

Two stroke phase models prevailed as the most commonly used across the included studies: the Chollet and Maglischo models. The most common was the Chollet framework with 11 studies (73%) using this model. The Maglischo framework was used in the three earliest studies (20%), with one additional study using a simplified entry and exit phase model (7%).

Of the 15 included studies, only four (27%) proposed a novel algorithm for phase segmentation [4,6,19,20]. The remaining 11 studies either adopted an existing approach with no changes (*n* = 7, 47%) or adopted an existing approach with some modifications (*n* = 4, 27%). Dadashi et al. [6] comprised the most influential methodological reference, with six subsequent studies (40%) citing it as the basis for segmentation.

Included studies used a variety of data signal features and processing methods for phase segmentation. The most common signal features were peak detection (*n* = 10, 67%), which identifies stroke phase events by locating local maxima or minima in the sensor signal; zero crossing detection (*n* = 3, 20%), which marks stroke phase events at points where the signal crosses zero; slope change detection (*n* = 2, 13%), which identifies transitions based on changes in the rate of signal change; and jerk detection (*n* = 2, 13%), which detects abrupt changes in acceleration. The most common data processing procedures were data filtering (*n* = 6, 40%), which reduces noise in the raw sensor signal, and orientation estimation (*n* = 3, 20%), which calculates the spatial orientation to provide a reference frame for phase detection. None of the included studies utilized any type of machine learning approach for phase segmentation; all relied exclusively on signal-based approaches. Three studies (20%) that adopted a prior algorithm without modification did not report any algorithmic details beyond citing the source method. Stroke phase segmentation and algorithm information is reported in Table 2.

### 3.5. Validation and Outcomes

**Figure 4:**
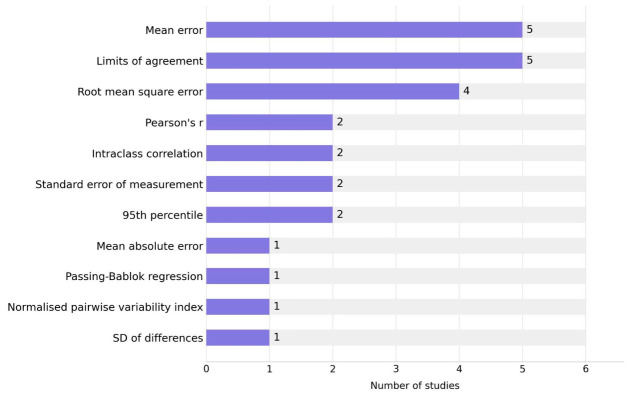
Algorithm validation metrics reported across included studies. All studies that validated their algorithm used video as the reference standard.

Six of the 15 included studies (40%) validated their phase detection algorithm, all using video as the reference standard. The remaining nine studies (60%) applied segmentation algorithms without reporting any validation of the phase detection itself. A variety of validation metrics were reported in the included studies, with the most common metrics being mean error and limits of agreement (*n* = 5 each, 33%). Information regarding the validation metrics reported can be found in Figure 4 (see Appendix A for full study validation details).

**Figure 5:**
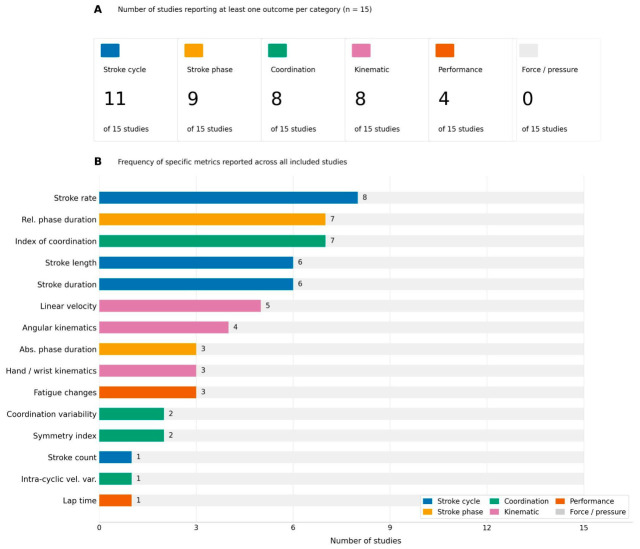
Outcome categories and specific metrics reported across included studies. (**A**) Number of studies reporting at least one outcome within each category. (**B**) Frequency with which each specific metric was reported across all 15 included studies, color-coded by outcome category.

Stroke rate (*n* = 8, 53%) and relative phase duration (*n* = 7, 47%) were the most consistently reported outcomes. IdC was the dominant coordination metric, reported across the majority of application-focused studies. Kinematic outcomes, including linear velocity, angular kinematics, and hand and wrist kinematics, were more common in earlier studies and those with a technology development focus. No study reported force or pressure outcomes of any kind. Outcome information is presented in Figure 5.

## 4. Discussion

The primary objective of this scoping review was to map the available evidence on sensor-based stroke phase segmentation methods in front crawl swimming, with specific attention to sensor characteristics, algorithmic approaches, validation strategies, and reported outcomes. The findings reveal a field that has undergone rapid methodological development since the early 2000s yet remains concentrated around a narrow set of approaches and outcomes, with several notable gaps that warrant future investigation.

The timeline of included studies reflects a field that is actively growing. The earliest work dates back to 2000, but 10 of the 15 included studies (67%) were published within the past decade. This suggests that wearable sensor-based phase segmentation in front crawl swimming has gained momentum in recent years. This growth can likely be attributed to broader trends in wearable technology development and analysis in swimming research [7].

The participant characteristics of the included studies raise questions regarding the generalizability of their phase segmentation algorithms. First, sample sizes ranged from *n* = 2 to *n* = 19. These small sample sizes limit statistical confidence in valid segmentation algorithms. Next, there was a wide range of competition levels represented in the studies, including elite, collegiate, national, regional, recreational, triathlon, and Paralympic. Only one study explicitly examined whether their segmentation algorithm performed consistently across populations, comparing algorithm performance between disabled and non-disabled swimmers [20]. Given that stroke kinematics can vary considerably across competition levels [20], algorithms validated on one population may not be generalizable to all populations. Future work should consider testing their algorithms on larger and more diverse sample sizes to determine the generalizability of their algorithms among broad populations.

A clear technological consensus has emerged across the literature: 11 of the 15 included studies (73%) employed IMUs for data collection. This shows a shift from the early work in the 2000s, where biaxial accelerometers were the primary sensors employed in the studies [4,9,10]. This convergence towards IMUs reflects both the development of technology over the decades, as well as its superiority in collecting the triaxial data inherent to front crawl swimming. The convergence also suggests the field has moved beyond initial sensor applications and towards a more standardized approach concerning device selection for phase segmentation. The dominance of IMUs in this context also has practical implications, as these smaller and wireless devices are more suitable in an ecologically valid environment than bulkier wired systems. However, IMUs are not without technological limitations in aquatic environments. Magnetometer accuracy can be affected by the ferromagnetic characteristics of pool infrastructure, resulting in a distorted magnetic field assumed by sensor fusion algorithms and potential errors in orientation estimation [14]. Additionally, attaching wearable sensors to swimmers has been shown to increase drag and significantly impact performance, potentially disrupting an ecologically valid environment [22].

The forearm emerged as the most common sensor placement, appearing in 13 of 15 included studies (87%), suggesting agreement on its utility for capturing the kinematic events necessary for stroke phase detection. This consistency is meaningful, as it suggests the forearm signal may be sufficiently reliable across a range of algorithmic approaches and study contexts. However, no included study directly compared phase detection quality across different sensor placements, leaving the question of optimal sensor location without a definitive answer. This is a significant gap, as sensor placement directly affects the signals used for phase segmentation. Additionally, this concentration has come at a cost, as it has excluded analysis of other important body segments. Most notably, the palmar surface of the hand was absent from sensor placement decisions across all included studies, despite being a critical surface responsible for forward propulsion in front crawl swimming [23]. This absence means that no study was positioned to capture pressure distribution or propulsive forces generated at the palm within each stroke phase.

The Chollet stroke phase framework was used in 11 of 15 studies (73%), establishing it as the dominant model in this area of research. The widespread use of this model allows comparability across studies and aligns with its capacity to quantify coordination metrics such as IdC. Notably, the Maglischo framework was only used in the three earliest studies and has not appeared in any work published after 2002. This suggests that the field made a deliberate shift toward the Chollet model early in its development. While the reasons for this shift are not explicitly documented in the included literature, it is possible that the Chollet model’s simpler four-phase structure may be more practical to implement within an algorithm; additionally, its direct link to coordination metrics such as IdC may make it a more analytically useful framework.

Of the data signal features examined in this review, peak detection emerged as the dominant approach, as it was employed in 10 of 15 studies (67%). This suggests that peak detection could be a reliable and practical method for identifying stroke phase events from wearable sensor signals. The most common processing procedures were data filtering and orientation estimation, reflecting a consistent method to deal with the practical challenges of utilizing sensor data in aquatic environments. These signal feature and data processing trends, along with the dominance of the Chollet model and IMU technology, point to a field that has established a reliable and reproducible technical foundation for sensor-based phase segmentation in front crawl swimming.

The absence of machine learning-based approaches in the included studies represents an area for growth in the field. Threshold-based approaches have proven to be useful, but inherently limit sensitivity in phase segmentation due to signal variability between swimmers [24]. Machine learning approaches have shown strong performance in IMU-based swimming analyses [24]. Whether similar advantages would extend to automatic phase segmentation represents a meaningful and unexplored direction in this field.

A significant methodological concern across the included studies is the absence of algorithm validation in the majority of included studies. Only six of the included studies validated their phase detection approach, all using video as the reference standard. The remaining nine studies adopted existing algorithms, most commonly that of Dadashi et al. [6], without reporting any validation of their own. While reliance on a common algorithm supports comparability across studies, adoption does not guarantee accurate implementation. Differences in sensor brand, sampling rate, placement precision, and swimmer population may all affect whether a previously validated algorithm performs accurately in a new context. Unvalidated adoption therefore risks both inaccurate phase detection and the propagation of the limitations inherent to the source algorithm. This raises concerns about the validity of phase detection, and by extension the downstream outcomes reported such as coordination metrics. For example, an error in identifying phase boundaries directly affects phase durations. This would corrupt coordination metrics such as IdC, where phase boundaries and durations are critical for calculation. Phase detection error could therefore produce misleading conclusions about swimmer coordination without any internal mechanism to detect the problem. Future work should treat algorithm validation as an important methodological standard to ensure accuracy of phase detection.

The sensor configurations reported in some studies also present practical concerns, with several studies employing upwards of five sensors across various body segments. This extensive setup would be logistically challenging outside of laboratory environments and limits the potential for application of this research in coaching contexts. Future research should therefore aim to achieve accurate phase detection using as few sensors as necessary to better support the application of this work in practical coaching settings.

The most consequential gap identified by this review is the complete absence of force and pressure outcomes across all 15 included studies. Current sensor-based segmentation methods are capable of identifying stroke phases, but provide no information about the mechanical demands experienced within those phases. This distinction is critical for applied practice; knowing that a swimmer spends a certain proportion of their stroke cycle in the pull phase offers a coach relatively little without understanding the propulsive forces generated during that phase. While prior work has examined intra-phase force contributions in front crawl swimming using camera-based three-dimensional kinematic analysis and hydrodynamic force estimation [3], this approach has not been extended to wearable sensor systems, representing a meaningful gap between laboratory-based biomechanical research and field-applicable technology. Compounding this issue is the absence of palmar sensor placements across the included literature. The hand is a critical propulsive surface in front crawl swimming [22], and the dorsal placements used in a small number of studies capture orientation and kinematic data but cannot characterize the pressure distribution experienced on the palmar surface during propulsion. The absence of palmar sensor placements in the literature is potentially attributable to the tendency of these sensors to add additional drag to the swimmer [23]. Despite this absence, pressure sensing systems integrated with IMUs have begun to emerge in the literature. Palmar-mounted devices have been used to quantify triaxial hand forces in ecologically valid environments [25]. This technology presents a meaningful opportunity to bridge the gap identified in this review, potentially enabling phase segmentation and intra-phase force analysis from a single wearable device. Integrating palmar pressure sensing represents a logical and necessary methodological advancement for a better understanding of optimal front crawl biomechanics.

### Limitations

As this is an emerging and heterogeneous area of research, critical appraisal of individual sources of evidence was not performed; this is consistent with scoping review methodology but means that findings from studies of varying methodological quality are treated equally. Additionally, this review did not examine the potential influence of sensor placement on swimmer biomechanics. Wearable sensors, particularly multi-sensor configurations spanning several body segments, may alter the kinematic or kinetic patterns that they are intended to measure [23], and this potential confound was not assessed as part of the present review. Also, the search in this review was limited to Web of Science, SPORTDiscus, and IEEE Xplore. Scopus was not searched in this review and may contain engineering-focused studies that were not captured in the current review.

## 5. Conclusions

This scoping review mapped the available literature on sensor-based stroke phase segmentation across 15 studies. The findings reveal a field that has converged methodologically around IMU-based approaches and the utilization of the Chollet model as a stroke phase framework. However, significant gaps remain in the literature. Algorithm validation was absent in the majority of included studies, and where validation was performed, approaches and metrics varied considerably, limiting the ability to draw comparisons across the literature. Most consequentially, no included study reported force or pressure outcomes of any kind, representing a critical disconnect between current sensor-based segmentation capabilities and the biomechanical information most relevant to understanding and optimizing front crawl performance.

Future research should address these gaps in two ways: first, algorithm validation should be more consistently prioritized. This will help to improve segmentation algorithms as well as ensure downstream outcomes reported are also accurate. Second, and most importantly, integrating force and pressure measurements into wearable-based phase segmentation represents a meaningful advancement that the field can make. The palmar surface of the hand remains entirely absent from sensor placement decisions across the literature despite being a critical propulsive surface in front crawl swimming. Incorporating palmar pressure sensors would allow researchers to characterize the mechanical demands generated within each phase. Addressing these gaps would move the field beyond temporal phase description toward a more complete, mechanistically grounded understanding of front crawl swimming.

## Figures and Tables

**Table 1:** Participant characteristics table.

Study	*n*	Sex	Age (Years)	Competition Level
[4]	2	Combined	N/R	Collegiate
[9]	5	Combined	N/R	Triathlon
[10]	5	Combined	N/R	Triathlon
[6]	7	Combined	18.7 ± 5.3	National
[11]	12	Combined	F: 17.0 ± 0.8; M: 19.0 ± 2.0	National
[12]	18	Combined	F: 19.3 ± 1.8; M: 16.0 ± 1.8	National and recreational
[13]	7	Male only	21.2 ± 2.96	National
[14]	6	Male only	26.1 ± 3.4	Regional
[15]	14	Male only	23.2 ± 2.8	National
[16]	8	Male only	20.8 ± 2.96	National
[17]	19	Combined	F: 21.7 ± 3.7; M: 20.7 ± 3.2	Elite
[18]	8	Male only	22.0 ± 2.5	National and regional
[19]	12	Male only	19.1 ± 2.3	Not reported
[20]	11	Combined	Non-disabled: F: 15 †; M: 19.2 ± 4.4. Disabled: F: 19.8 ± 2.1; M: 36 †	Paralympic (disabled group); not reported (non-disabled group)
[21]	15	Combined	F: 20.1 ± 2.3; M: 22.9 ± 6.7	Elite and Paralympic

Note: studies sorted chronologically. *n* = number of participants; F = female; M = male; N/R = not reported. † Single participant; no SD applicable.

**Table 2:** Sensor and algorithm characteristics.

Study	Sensor Type	Name/Brand	Freq (Hz)	Sensors (n)	Placement	Phase Model	Algorithm Approaches	Algorithm Status	Cites
[4]	Accel (biaxial)	ADXL250, Analog Devices	N/R	2	Wrist	Maglischo	Peak detection; Zero crossing	Novel	N/A
[9]	Accel (biaxial)	ADXL210, Analog Devices	128	2	Forearm	Maglischo	Peak detection; Zero crossing	Adopted	[4]
[10]	Accel (biaxial)	ADXL210, Analog Devices	128	2	Forearm	Maglischo	Peak detection	Adopted	[4]
[6]	IMU; Accel (triaxial); Gyroscope	Physilog III, BioAGM	500	3	Forearm; Lower back	Chollet	Peak detection; Slope change detection; Orientation estimation; Filtering	Novel	N/A
[11]	Accel (triaxial)	X6-2mini, Gulf Coast Data Concepts	320	6	Arm; Wrist; Upper back; Lower back	Chollet	Peak detection; Zero crossing	Adopted with changes	[10]
[12]	IMU; Accel (triaxial); Gyroscope	Physilog III, BioAGM	500	3	Forearm; Lower back	Chollet	N/R	Adopted	[6]
[13]	IMU; Accel (triaxial); Gyroscope; Magnetometer	HIKOB Fox, Hikob	100	7	Arm; Forearm; Hand (dorsal); Lower back	Chollet	Peak detection; Orientation estimation; Filtering	Adopted with changes	[6]
[14]	IMU; Accel (triaxial); Gyroscope; Magnetometer	APDM OPAL, APDM	128	7	Arm; Forearm; Hand (dorsal); Thorax/chest	Chollet	Orientation estimation; Filtering	Adopted	[6]
[15]	IMU; Accel (triaxial); Gyroscope; Magnetometer	APDM Opal, APDM	128	5	Arm; Forearm; Thorax/chest	Chollet	Peak detection; Threshold detection; Filtering	Adopted with changes	[6]
[16]	IMU; Accel (triaxial); Gyroscope; Magnetometer	HIKOB Fox, Hikob	100	7	Arm; Forearm; Hand (dorsal); Lower back	Chollet	N/R	Adopted	[13]
[17]	IMU; Accel (triaxial); Gyroscope; Magnetometer	HIKOB Fox, Hikob	100	3	Forearm; Lower back	Chollet	Peak detection	Adopted with changes	[6,13]
[18]	IMU; Accel (triaxial); Gyroscope; Magnetometer	APDM Opal, APDM	128	5	Arm; Forearm; Thorax/chest	Chollet	N/R	Adopted	[15]
[19]	IMU; Accel (triaxial); Gyroscope	Model not reported, Cometa	285	5	Head; Forearm; Ankle	Other: entry/exit	Peak detection; Jerk	Novel	N/A
[20]	IMU; Accel (triaxial); Gyroscope; Magnetometer	Wavetrack, Cometa	2000	2	Forearm	Chollet	Peak detection; Slope change detection; Jerk; Filtering	Novel	N/A
[21]	IMU; Accel (triaxial); Gyroscope; Magnetometer	Wavetrack, Cometa	2000	5	Forearm; Lower back; Leg	Chollet	Filtering	Adopted	[6,20]

Note: Accel = accelerometer; Freq = sampling frequency; N/A = not applicable; N/R = not reported. Algorithm status: Novel = algorithm developed by the study authors; Adopted = prior algorithm applied without modification; Adopted with changes = prior algorithm applied with modifications.

## Data Availability

No new data were created or analyzed in this study. Data sharing is not applicable to this article.

## Supplementary Materials

The following supporting information can be downloaded at https://www.mdpi.com/article/10.3390/s26134221/s1, File S1: Preferred Reporting Items for Systematic reviews and Meta-Analyses extension for Scoping Reviews (PRISMA-ScR) Checklist.

## Appendix A

**Table A1:** Algorithm validation information.

Study	Method	Validation Metrics
[4] ^†^	Video	Qualitative cross-referencing only; no quantitative metrics reported
[6]	Video	Mean error Limits of agreement (Bland–Altman) Intraclass correlation coefficient Passing–Bablok regression Standard deviation of difference Normalised pairwise variability index
[11]	Video	Mean error Mean absolute error Root mean square error Standard error of measurement Limits of agreement (Bland–Altman) 95th percentile for absolute error
[15]	Video	Mean error Root mean square error Limits of agreement (Bland–Altman) Pearson’s r correlation Intraclass correlation coefficient
[19]	Video	Mean error Root mean square error Limits of agreement (Bland–Altman) Pearson’s r correlation 95th percentile for absolute error
[20]	Video	Mean error Root mean square error Standard error of measurement Limits of agreement (Bland–Altman)

Note: Nine studies did not validate their phase detection approach and are not included in this table. ^†^ Ohgi et al. [4] cross-referenced acceleration signal features with video-derived hand paths qualitatively; no quantitative agreement metrics were reported. This represents a lower level of validation than the remaining five studies.
